# The prospects of flexible natural gas-fired CCGT within a green taxonomy

**DOI:** 10.1016/j.isci.2023.107382

**Published:** 2023-07-14

**Authors:** Mai Bui, Nixon Sunny, Niall Mac Dowell

**Affiliations:** 1 Centre for Environmental Policy, Imperial College London, London, UK; 2 Centre for Process Systems Engineering, Imperial College London, London, UK

**Keywords:** Energy policy, Energy Modelling, Energy Systems

## Abstract

Despite increased commitments toward net zero, there will likely be a continued need for natural gas to provide low carbon dispatchable power and blue hydrogen to balance the increased penetration of renewables. We evaluate the CO_2_ emissions intensity of electricity produced by (i) natural gas-fired combined cycle gas turbine (CCGT) power plants with carbon capture and storage (CCS), and (ii) blue hydrogen CCGT plants which uses steam methane reforming with CCS to supply H_2_. This study aims to determine whether these assets are able to meet a possible green taxonomy emissions threshold of 100 kg CO_2_ eq/MWh. Key considerations include methane leakage, CO_2_ capture rate, and the impacts of start-up and shut down cycles performed by the CCGT-CCS plant. This study suggests that, in order for natural gas to play an enduring role in the transition toward net zero, managing GHG emissions from both the upstream natural gas supply chain and the conversion facility is key.

## Highlights

• The green taxonomy has an emissions threshold of 100 kg/MWh
• Under a green taxonomy, managing gas supply chain emissions and the CO_2_ capture facility is a key
• For flexible gas CCGT-CCS, hot SUSD can be frequent, whereas cold SUSD cycles will need to be limited
• Blue hydrogen-CCGT can provide a high degree of flexibility without increasing CO_2_ emissions

## Graphical abstract

**Figure:**
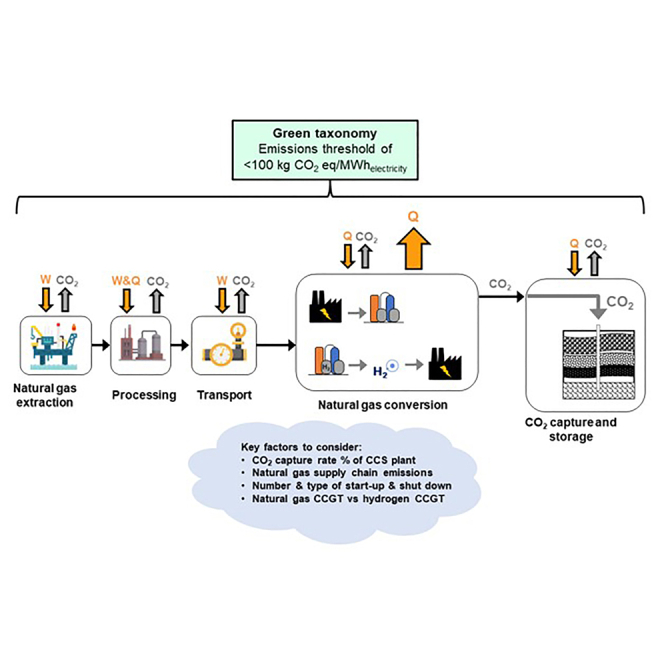

## Introduction

### Reducing GHG emissions across supply chains

In order to limit global warming to 1.5°C, immediate and rapid reduction of greenhouse gas (GHG) emissions is essential.^1^^,^^2^ However, natural gas (NG) is anticipated to retain an important role in the transition to a decarbonized global energy system.^3^ Short-term disruption to international gas supplies notwithstanding, in the medium to long term, NG is expected to continue its existing role in replacing coal for electricity generation.^3^^,^^4^ Combining gas-fired power plants with carbon capture and storage (CCS) can provide affordable, dispatchable, low carbon capacity, which will have a role in maintaining security of supply and enabling the expansion of other low-carbon sources.^5^^,^^6^^,^^7^^,^^8^^,^^9^ In the long-term, NG will remain an important feedstock for the large-scale production of hydrogen,^10^^,^^11^^,^^12^^,^^13^ with potential applications in decarbonizing transport,^14^ heat,^12^ and power.^15^^,^^16^

**Figure 1:**
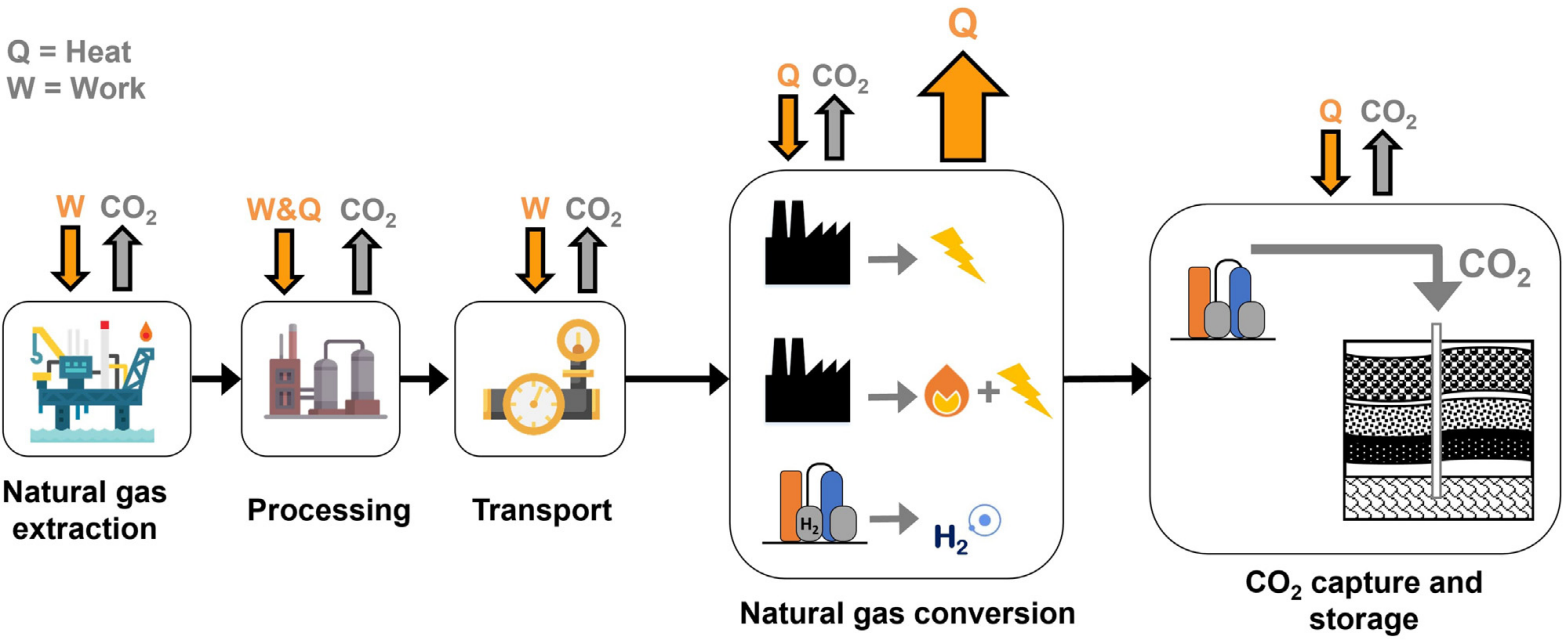
The distribution of GHG and CO_2_ emissions along the natural gas value chain

Regardless of the application for NG, quantifying its supply chain emissions has become an increasingly important topic as climate targets become more stringent.^3^^,^^17^^,^^18^^,^^19^ However, this only represents the emissions at a single point in the value chain of the fossil fuel, which may have been extracted elsewhere and used to provide goods or services in a different location (Figure 1). Therefore, understanding the distribution of GHG emissions along entire supply chains (e.g., fossil fuels, biomass) will help facilitate international efforts to limit CO_2_ emissions.^20^

To measure and manage emissions more consistently, the Greenhouse Gas (GHG) Protocol established comprehensive standardized frameworks for GHG emissions reporting. Since the release of the first edition in 2001, it has since been updated with an additional guidance on how to measure and account for emissions throughout the value chains. It has become the most widely used GHG accounting standards for measuring and reporting GHG emissions,^21^^,^^22^ and is used by major NG producers such as bp,^23^ Shell,^24^ TotalEnergies,^25^ and Chevron.^26^ Recent studies have demonstrated the importance of considering supply chain emissions. In the case of NG, accounting for the supply chain emissions has been shown have a significant impact on the overall CO_2_ reduction potential of CCS used for hydrogen production^27^^,^^28^ and power/heat generation.^29^ As more countries commit to net zero emission targets, understanding the implications of international supply chain emissions will be important in quantifying the techno-economic burden of decarbonization pathways and policy.

### The importance of flexible CCS in the energy transition

When considering plants with CCS in the context of net zero targets, higher capture rates of 95–99% are recognized as essential.^30^^,^^31^^,^^32^^,^^33^^,^^34^^,^^35^ Studies that have considered higher capture rates to date are based on steady state operation. However, the high integration of intermittent renewables (e.g., wind, solar photovoltaics) in future energy systems presents major operational challenges^36^^,^^37^ and will require greater system flexibility.^38^ As the penetration of intermittent renewables increases, the frequency of start-up and shut down (SUSD) cycles of power plants with CCS is also expected to increase.^39^^,^^40^ Evidence in the literature indicates that achieving high CO_2_ capture rates above 90% may be challenging during SUSD,^41^^,^^42^^,^^43^^,^^44^ especially with cold start-up and shut down cyles.^44^ If the CO_2_ emissions increase considerably during SUSD, this could undermine the value proposition of CCS as a flexible, low carbon asset. While the value of flexible operation in terms of load following and part-load operation has been demonstrated in previous studies, there has been limited work to date that focuses on the impact of start-up and shutdown on CO_2_ capture plants. Work is therefore necessary to understand the impact of start-up and shutdown on the CO_2_ emissions of power plants with CCS.^44^

### Green taxonomy: Supporting net zero energy transition

A sustainable, or green, taxonomy, provides financial firms a common classification framework for: (i) managing their exposure to climate risk, and (ii) **making informed investment decisions** on sustainable economic activities.^45^^,^^46^ It establishes clarity on which activities can qualify as “green” and address social issues.^46^ The regulation imposes a mandatory reporting obligation on certain companies and investors, requiring them to disclose the share of their taxonomy-aligned activities and investments. Although meeting the taxonomy criteria are not mandatory, the obligation of transparent reporting provides a motivation for companies to improve their environmental performance. The need to be recognized as “green” and sustainable will become increasingly important in attracting investment as financial markets transition toward carbon neutrality.^47^

The European Union (EU) Taxonomy can be used by any company/investor to classify their economic activities as sustainable or green. However, under the Taxonomy, reporting will be a **mandatory** requirement for: (1) financial market participants and issuers offering financial products within the EU, including the UK; (2) large companies with over 500 employees that are already required to provide a non-financial statement under the EU Non-Financial Reporting Directive (NFRD); and (3) EU and Member States when they are setting public measures, standards or labels for green financial products or green bonds.^47^^,^^48^

The EU Taxonomy regulation framework translates environmental and sustainability policy goals into a framework, providing a list of economic activities that are able to substantially contribute to climate change mitigation while minimizing environmental harm.^49^^,^^50^ The key environmental objectives are climate change mitigation, adaptation, protection of water, ecosystems, circular economy, and tackling pollution.^51^^,^^52^ The taxonomy covers a range of activities including agriculture, forestry, manufacturing, energy, transportation, etc.^53^ The taxonomy proposes an overarching, technology-agnostic emissions threshold of 100 kg CO_2_ eq/MWh for electricity generation, heat production, and co-generation of heat and electricity. Starting from 2020, this threshold will reduce every five years in line with the trajectory of government targets to achieve net-zero CO_2_eq by 2050.^49^^,^^50^ To prove eligibility, a product carbon footprint (PCF) assessment that is compliant with ISO 14067 or a GHG Protocol Product Life cycle Standard is required. Furthermore, the assessment should include life cycle fugitive emissions (i.e., methane leakage between the point of extraction to the energy production site) from actual physical measurements rather than estimates.^51^

For hydrogen production within the EU taxonomy, the life cycle GHG emissions threshold needs to be lower than 3 tCO_2_ eq/t H_2_. This threshold favors green hydrogen, however, low carbon blue hydrogen (e.g., steam methane reforming with CCS) and turquoise hydrogen (i.e., methane pyrolysis to produce hydrogen and solid carbon) can also meet taxonomy criteria.^54^

**Figure 2:**
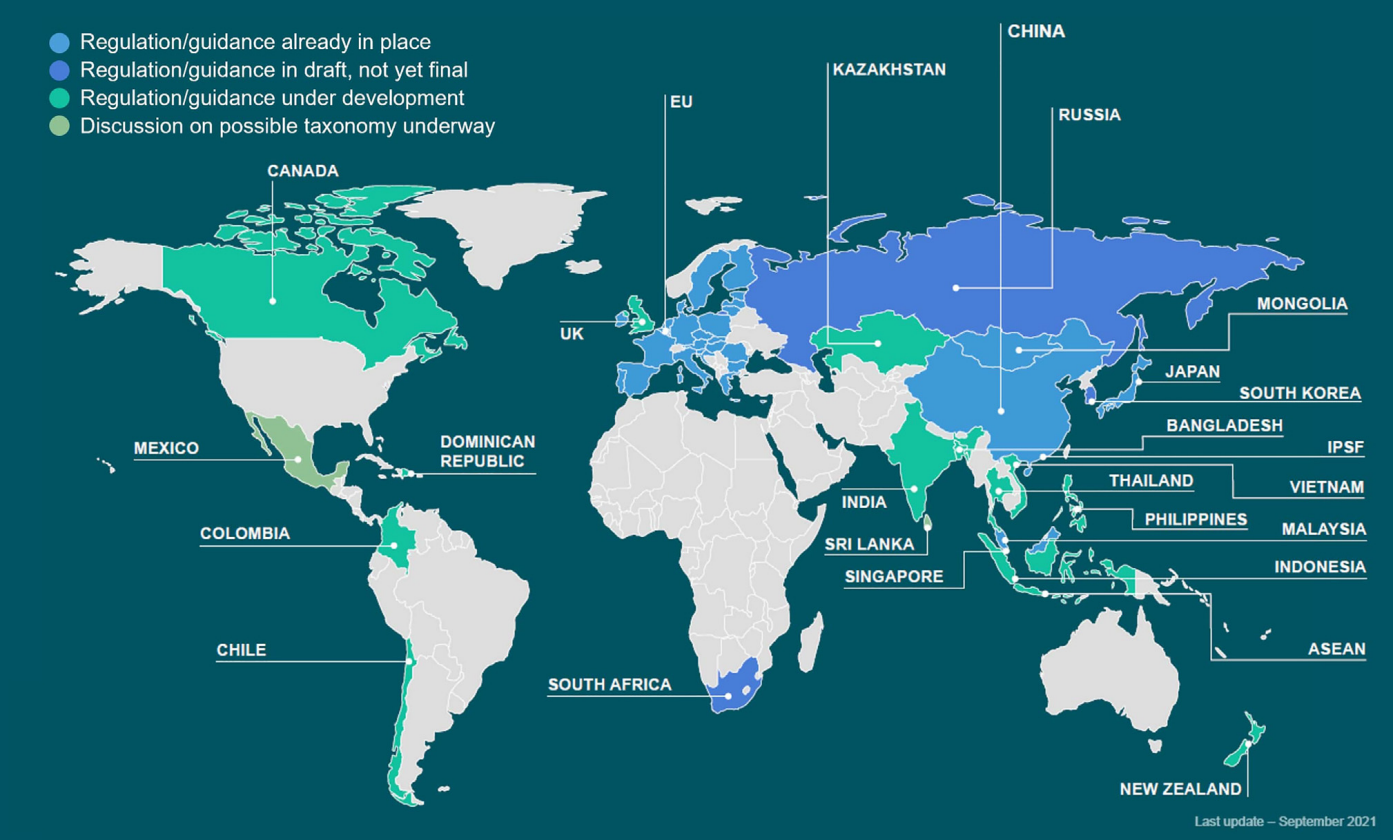
Status of sustainable taxonomy development around the world Figure adapted from FoSDA.^55^ Gray colored regions have not yet considered the development of a sustainable taxonomy.

Aside from the EU, other countries that have taxonomy regulation in place include China, Japan, Malaysia, and Mongolia, with many other jurisdictions having taxonomy regulation being drafted or under development^55^ as shown in Figure 2. Although there is some heterogeneity in the approaches used to develop and implement taxonomies across different jurisdictions, many countries are using the EU Taxonomy as a benchmark. For example, South Africa, South Korea, Canada, and the UK have closely aligned or drawn inspiration from the EU Taxonomy. Some countries such as Russia and Sri Lanka are modeling a taxonomy based on both the Chinese and EU taxonomies.^46^^,^^55^ Similarly, the US is also expected to leverage the EU taxonomy in designing their framework given that there has already been a considerable level of engagement between US and EU regulators on climate change, taxonomies, and standards.^45^ Based on the current trends thus far, it seems highly likely that any sustainable taxonomy developed in the future by additional countries will mirror the EU Taxonomy.

Under the EU Taxonomy framework, a near-term emissions threshold of 100 kg CO_2_ eq/MWh will have significant implications on the energy sector. For instance, any unabated fossil fuel-fired power plants (i.e., without CCS) will not meet the required carbon intensity threshold. Even with CCS, coal-fired power plants are unlikely to meet the threshold in the long-term, owing to the reduction in the emissions threshold which will reach zero kg CO_2_ eq/MWh by 2050. In contrast, NG-fired power plant with CCS has the potential to qualify, however, eligibility will be subject to the levels of fugitive emissions along the NG supply chain and the CO_2_ capture rate of CCS.^49^^,^^56^

### Study objectives

To make a substantial contribution to the Paris Agreement targets within the sustainable taxonomy, the EU Technical Expert Group recommends that a power generator operates below 100 kg CO_2_ eq/MWh_el_ over its lifetime.^49^^,^^50^ In contrast, operating power plants at a carbon emissions intensity above 270 kg CO_2_ eq/MWh_el_ will make meeting mitigation target very challenging.^56^

To determine the eligibility of a combined cycle gas turbine (CCGT) power plants within any future sustainable green taxonomy, it will be essential to understand the effect of key factors on the carbon intensity of the electricity generated. The study aims to demonstrate the impact of key process factors on the CO_2_ intensity of the electricity generation by a CCGT power plant. The factors that will be evaluated include.

(1) Natural gas supply chain emissions (Scope 3);
(2) CO_2_ capture rate;
(3) Switching to blue hydrogen;
(4) Start-up and shut down cycles.

The objective of the analysis is to identify potential constraints for each of these factors when considering a sustainable taxonomy with an emissions threshold of < 100 kg CO_2_ eq/MWh_el_ for electricity generation.^49^^,^^50^

## Results

### Impact of supply chain emissions and CO_2_ capture rate

**Figure 3:**
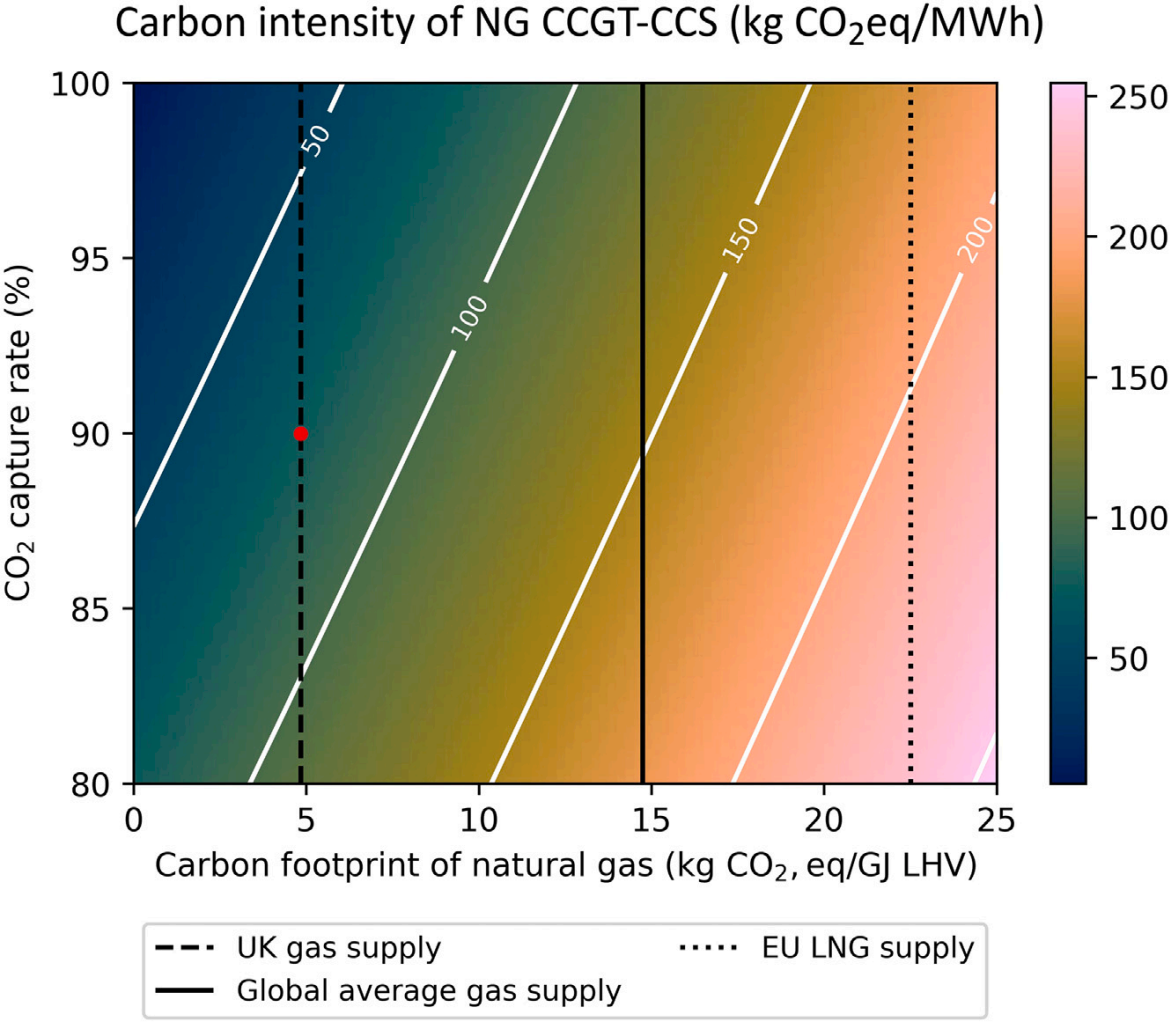
CO_2_ intensity of a natural gas-fired CCGT-CCS plant as a function of the carbon footprint of the natural gas and the CO_2_ capture rate The analysis assumes steady state operation and a natural gas-fired CCGT power plant with an unabated efficiency of 58% with an energy penalty for CO_2_ capture. The red point corresponds to CCGT-CCS using UK gas supply with 90% capture (75.2 kg CO_2_ eq/MWh_el_).

**Figure 4:**
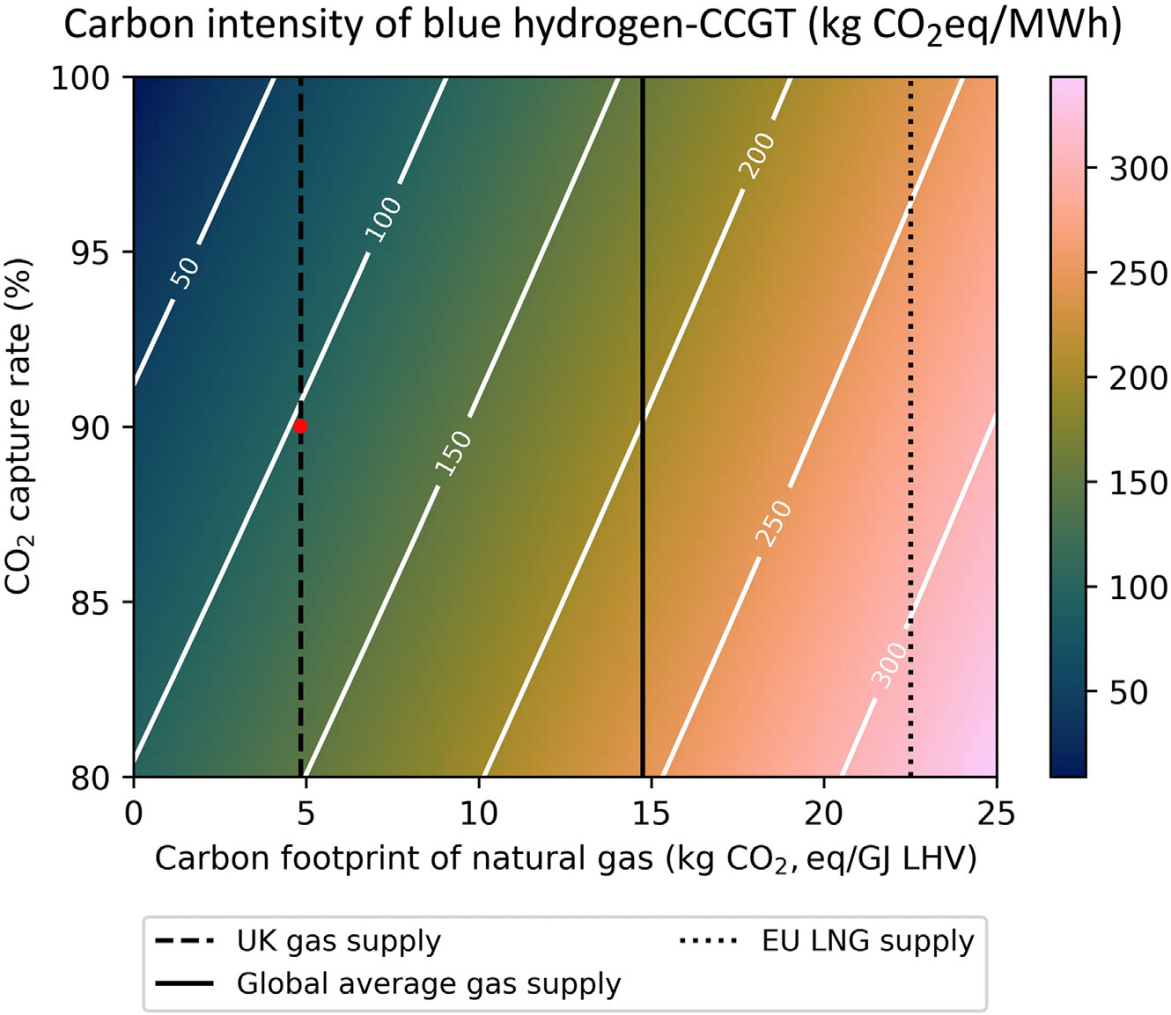
CO_2_ intensity of a blue hydrogen-fired CCGT-CCS plant (kg CO_2_ eq/MWh_el_) as a function of the carbon footprint of the natural gas used for hydrogen production and the CO_2_ capture rate of SMR The analysis assumes steady state operation and considers an SMR plant retrofitted with CO_2_ capture, providing blue hydrogen for a CCGT power plant with an efficiency of 58%.

The fugitive emissions along supply chains can vary, resulting in different NG carbon footprints. The vertical lines in Figures 3 and 4 correspond to the carbon footprints of NG from the UK,^57^ global average,^58^ and EU liquid natural gas (LNG) supply^59^—data summarized in Table S1. As shown in Figure 3, the carbon emissions intensity of a NG-fired CCGT power plant decreases with lower carbon footprint of the NG and higher CO_2_ capture rate. The same trend is observed with the carbon emissions intensity of a CCGT power plant firing blue hydrogen produced from SMR retrofitted with CCS (Figure 4).

Importantly, using NG with the global average carbon footprint in both the gas CCGT-CCS and blue hydrogen CCGT systems exceeded the taxonomy emissions threshold, regardless of the CO_2_ capture rate. Hence, these technologies would only align with a green taxonomy if the integrity of global NG supply chains is tightened further to reduce fugitive GHG emissions (e.g., 5–8 kg CO_2_/GJ LHV).

At a given NG carbon footprint and CO_2_ capture rate, the hydrogen CCGT power plant has a higher CO_2_ intensity compared to the NG CCGT plant (Figures 3 and 4). Using a UK gas supply in the CCGT-CCS plant with an average 90% CO_2_ capture achieves a CO_2_ intensity of 75.2 kg CO_2_ eq/MWh_el_. This indicates that NG-fired CCGT-CCS plants are able to satisfy the requirements of a sustainable taxonomy.

In contrast, using a UK gas supply for SMR retrofitted with CCS and capturing 90% CO_2_ only delivers 103 kg CO_2_ eq/MWh_el_ of CO_2_ intensity. As discussed in a previous contribution, scope 3 supply chain emissions (x axis) attributed to blue hydrogen production (i.e., SMR with CCS) are higher compared to those of NG.^29^ Consequently, the blue hydrogen-CCGT plant using SMR with 90% CO_2_ capture is unable to achieve a carbon intensity that aligns with the EU taxonomy, i.e., below 100 kg CO_2_ eq/MWh_el_. To achieve a CO_2_ intensity that satisfies the taxonomy, the blue hydrogen-CCGT plant would need to either use (i) CO_2_ capture rates higher than 91% to produce blue hydrogen from SMR, (ii) NG with low supply chain emissions (e.g., UK NG), or (iii) green hydrogen derived electrolytically using renewable energy (i.e., with lower carbon footprint). For instance, assuming a CO_2_ capture rate of 95%, a blue hydrogen-CCGT could deliver power at a carbon intensity of 80 kg CO_2_ eq/MWh_el_.

### Will flexible operation be possible within a sustainable taxonomy?

**Figure 5:**
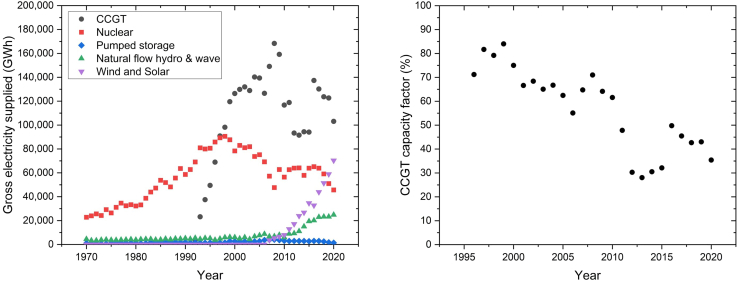
Representative load factors of gas-fired power plants in the UK (Left) Yearly gross electricity supplied in the UK from 1970€ to 2020. (Right) Yearly average plant capacity factor, i.e., load factor, of combined cycle gas turbine (CCGT) power plants in the UK starting from 1996 up until 2020. Data Source: UK National Statistics.^60^

In the previous section, burning NG in a CCGT plant with CCS was found to be more effective than blue hydrogen CCGT at reducing CO_2_ emissions, which concurs with Sunny, et al.^29^ However, those results assume steady state operation of the CCGT-CCS system. At steady state, plant conditions are typically optimal and can achieve relatively high CO_2_ capture rates. However, as shown by Figure 5, the average annual capacity factor of CCGT power plants in the UK has been declining. The initial years of CCGT deployment had higher capacity factors, ranging between 71 and 84%, which indicate CCGT mostly operated as baseload capacity. As the supply of intermittent renewables increased, the annual capacity factor of CCGT decreased to 28–50% (between 2011 and 2020),^60^ which corresponds to increased flexible operation of UK CCGT plants. Thus, CCS integrated in CCGT plants would likely need to operate flexibly as well.^61^

Highly flexible operation such as start-up and shut down (SUSD) cycles of power plants with CCS imposes significant process disturbances and causes the system to deviate away from optimal conditions. As a result, the residual CO_2_ emissions will increase, which in turn reduces the cumulative CO_2_ capture rate of the process.^41^^,^^42^^,^^43^^,^^44^^,^^62^^,^^63^

During full load, steady state operation of the power plant, steam for the CO_2_ capture process is supplied via steam extraction between the IP and LP turbines of the power plant steam cycle.^42^^,^^64^ However, steam extraction is not possible during start-up and shut down of the power plant.^43^ Consequently, when steam extraction from the power plant is unavailable, a heater or auxiliary boiler may be used to provide steam during SUSD of the CO_2_ capture process.^65^^,^^66^ Depending on the fuel or energy type, the use of an auxiliary boiler will have an associated CO_2_ emissions penalty.^44^

**Figure 6:**
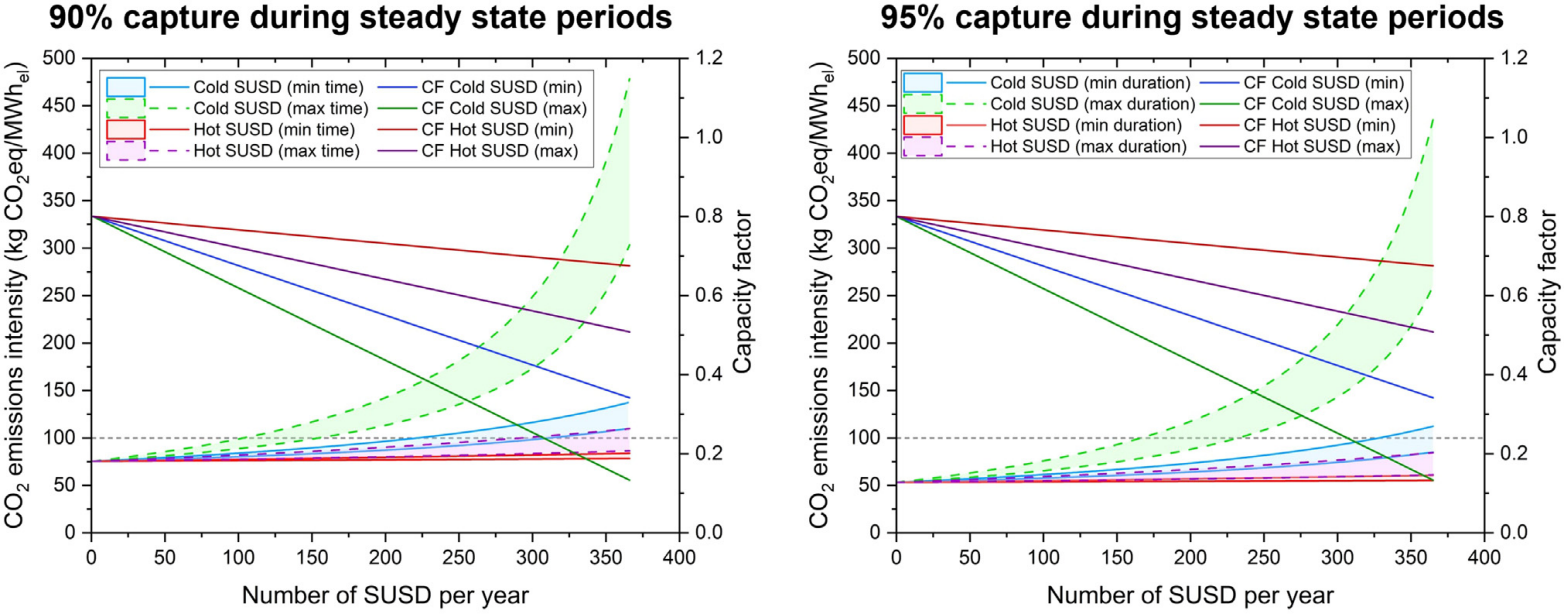
Effect of increasing start-up and shut down (SUSD) cycles on the CO_2_ emissions intensity and capacity factor of a CCGT power plant with CCS, assuming 90% capture rate (left), and 95% capture rate (right) The CO_2_ emissions intensity of the plant is a function of type of start-up (cold vs. hot) and the duration (min vs. max times). For the CO_2_ emissions intensity of a given number of SUSD cycles, the lower bound corresponds to SUSD with an auxiliary boiler that has zero CO_2_ intensity (e.g., renewable energy), whereas upper bound represents SUSD with a natural gas-fired auxiliary boiler. The CO_2_ emissions intensity account for both Scope 1 (direct combustion) and Scope 3 (supply chain) emissions. Results for SUSD with 99% capture rate at steady state are also provided in Figure S5.

The CO_2_ emissions intensity of the NG-fired CCGT in Figure 6 considers both Scope 1 (direct combustion) and Scope 3 (supply chain) emissions. For the CO_2_ emissions intensity of a given number of SUSD cycles, the lower bound corresponds to SUSD with an auxiliary boiler that has zero CO_2_ intensity (e.g., renewable energy), whereas upper bound represents SUSD with a NG-fired auxiliary boiler. In Figure 6, as the number of start-up and shut down cycles of the natural-gas-fired CCGT-CCS plant increases, the CO_2_ emissions intensity becomes increasingly higher, and the capacity factor reduces. Capacity factor is a function of the number of start-up and shut down cycles, as well as the type (cold vs. hot) and duration (min vs. max). At the higher numbers of start-up and shut down cycles, the capacity factors for most of the SUSD cases are in line with the annual averages observed for CCGT operation in the UK (Figure 5), with the exception of the cold SUSD at max duration.

The CO_2_ capture performance can vary depending on the start-up and shut down type, duration (Table S4) and the CO_2_ capture rate used for the full load steady state periods (Table S5). For cold SUSD cycles, the CO_2_ emissions intensity increases significantly compared to hot start-up and shut down cycles. As shown in Figure 6, hot SUSD cycles are able to meet the requirements of a sustainable taxonomy. In contrast, there needs to be a constraint on the number of cold SUSD cycles to limit the CO_2_ emissions intensity below 100 kg CO_2_ eq/MWh_el_. If a higher CO_2_ capture rate (95–99%) is used for the steady state periods, a larger number of cold SUSD cycles are possible (Table S6, owing to the lower level of residual CO_2_ emissions.

There are gas turbines capable of firing up to 100 vol % hydrogen already commercially available, e.g., B/E-class and F-class gas turbines by GE,^67^ with other companies aiming to develop 100% H_2_ gas turbines by 2030.^68^^,^^69^ Recent studies indicate that hydrogen-fired CCGT power plants are potentially as flexible as NG-fired CCGT plants.^69^^,^^70^^,^^71^^,^^72^ Therefore, hydrogen-fired CCGT power plants may also have a role in providing dispatchable low carbon electricity, e.g., as shown by CCC^73^ for the UK electricity grid. However, the hydrogen fuel needs to be highly carbon efficient in order to meet the EU taxonomy, e.g., blue hydrogen using UK gas with CO_2_ capture rates > 91% or green hydrogen (Figure 4). Unlike a NG-fired CCGT with CCS, the main advantage of H_2_-fired CCGT is that SUSD and highly flexible operation will not increase the CO_2_ emissions intensity of the electricity. Thus, the type and number of start-up and shut down cycles in H_2_-CCGT plants would not be constrained.

## Discussion

### Meeting the green taxonomy carbon emissions threshold

Although meeting the EU taxonomy criteria are not mandatory, the regulation has a mandatory reporting obligation for large companies, requiring them to disclose the share of their Taxonomy-aligned activities, thus, impacting investment potential.^49^^,^^50^ A sustainable, or green, taxonomy is a regulation framework which provides financial firms a common classification to manage their exposure to climate risk, and make informed investment decisions on sustainable economic activities. Understanding the potential impact of a green taxonomy on the prospects of existing and future fossil fuel-based power generation is essential in ensuring a cost-effective transition to net zero.

With higher penetration of intermittent renewable energy, thermal power plants with CCS could have a crucial role in providing low carbon, dispatchable electricity.^9^^,^^36^^,^^37^^,^^38^^,^^74^ Energy systems modeling show that as the UK energy system evolves and transitions to net zero by 2050, thermal power plants with CCS will likely operate as a peaking plant instead of baseload.^6^ However, studies at the systems level often assume a constant CO_2_ capture rate of 90% for CCGT-CCS. There is growing evidence indicating that highly flexible operation of CCGT-CCS plants including start-up and shut down cycles can lead to reduced CO_2_ capture rates, resulting in higher residual CO_2_ emissions.^41^^,^^42^^,^^43^^,^^44^ The use of NG as a fuel also contributes to the CO_2_ intensity of the electricity, owing to the fugitive emissions associated with the NGsupply chain. Therefore, using NG with a high carbon footprint can also increase the CO_2_ emissions intensity significantly. The sustainable taxonomy provides a technology agnostic threshold on CO_2_ emissions intensity; however, the taxonomy does not provide guidance on how the asset should be operated over its lifetime. The specific constraints that arise as a consequence of this threshold are still unclear, e.g., limits in operating window, operation modes, NG carbon footprint, CO_2_ capture rates.

This work evaluates the CO_2_ emissions intensity of electricity produced by (i) NG-fired CCGT power plants with CCS, and (ii) blue hydrogen CCGT plants which uses SMR with CCS to supply H_2_. The effects of key factors such as Scope 3 NG supply chain emissions, CO_2_ capture rate and the number of SUSD cycles were evaluated, thereby identifying constraints required to meet a sustainable taxonomy emissions threshold of < 100 kg CO_2_ eq/MWh.

For a NG-fired CCGT-CCS assuming steady state operation, using NG with a UK carbon footprint (4.9 kg CO_2_/GJ LHV) and a CO_2_ capture rate > 83% will meet the requirements for a sustainable taxonomy. However, the taxonomy CO_2_ intensity criteria become infeasible for higher NG carbon footprint and CO_2_ capture rates below 83%. The constraints for NG supply chain emissions and CO_2_ capture rate become stricter when considering blue H_2_ fired-CCGT. Assuming the use of UK NG for blue hydrogen-fired CCGT required the CO_2_ capture from the SMR to be 91% or higher, whereas 90% CO_2_ capture was unable to satisfy the taxonomy requirements.

On a steady state basis, the NG-CCGT with CCS performed better than the H_2_-CCGT in terms of CO_2_ emissions intensity. However, if we consider plants that operate more flexibly (e.g., at capacity factors < 0.5), the CCGT-CCS plant conditions deviate from its optimal conditions. Consequently, the CO_2_ emissions intensity of NG-fired CCGT-CCS plants increase significantly as a function of plant flexibility (i.e., type of SUSD and the number of SUSD cycles). Under the assumption that 95% CO_2_ capture rates are used during steady state periods, i.e., UK best available techniques guidance,^35^ we demonstrate that NG-fired CCGT-CCS plants are not limited by the number of hot SUSD cycles and should be able to meet the requirements of a sustainable green taxonomy. Under the taxonomy, the worse-case scenario of cold SUSD and 95% steady state capture will be limited to 166 cycles for a NG auxiliary boiler, and up to 232 cycles with a zero-carbon auxiliary boiler (Table S6). Improving the cold SUSD protocol (i.e., reducing the duration and using a zero-carbon auxiliary boiler) will not have a limit on the number of SUSD cycles. Conversely, cold SUSD using a lower 90% capture rate further limits the number of SUSD cycles that can be performed annually. Therefore, employing higher CO_2_ capture rates during periods of steady state enables greater flexibility (i.e., increases the number of allowable SUSD cycles) within the sustainable taxonomy.

Alternatively, H_2_-fired CCGT can also provide low-carbon, dispatchable electricity and is as flexible as NG-CCGT plants.^69^^,^^70^^,^^71^^,^^72^ The main benefit is the H_2_-fired CCGT is that the CO_2_ emissions intensity of the electricity is mainly a function of the NG carbon footprint and the CO_2_ capture rate in the hydrogen production process. Flexible operation of H_2_-CCGT does not increase CO_2_ eq emissions, assuming steady state operation of the hydrogen production facility. Thus, the H_2_-CCGT could provide a high degree of flexibility to the energy system without increasing CO_2_ emissions intensity. However, to meet the taxonomy requirements, the hydrogen will need to be produced from low carbon footprint NG (e.g., 4.9 kg CO_2_/GJ LHV) and use high capture rates (i.e., greater than 91%). Another important consideration is that a large number of SUSD cycles would likely negatively impact the overall power efficiency of the H_2_-fired CCGT, similar to the case of high SUSD cycles in NG-fired CCGTs.

The 100 kg CO_2_ eq/MWh emissions threshold is the *near-term* green classification for electricity generation. This taxonomy threshold will reduce every five years and will eventually reach net-zero CO_2_eq by 2050.^49^^,^^50^ Therefore, to ensure that gas CCGT-CCS and blue hydrogen-CCGT systems remain in line with the net-zero taxonomy requirements in the long term, the integrity of NG supply chains would need to tighten to reduce fugitive GHG emissions further. Additionally, higher CO_2_ capture rate would need to be employed. For instance, the upper limit CO_2_ capture rate for post-combustion capture of 95–99% should be used,^30^^,^^31^^,^^32^^,^^33^^,^^34^^,^^35^ whereas CO_2_ capture in blue hydrogen production has an upper limit of 90–95%.^11^^,^^75^^,^^76^ Even if the lowest feasible CO_2_ emissions intensity is achieved, gas CCGT-CCS and blue hydrogen-CCGT systems would have some degree of residual CO_2_ emissions. Thus, CO_2_ removal from the atmosphere would be required to offset these residual emissions and meet the net-zero CO_2_ eq threshold in 2050.

Lastly, the influence of flexible operation (e.g., SUSD cycles) must also be considered when determining whether the taxonomy criteria have been met. The residual CO_2_ eq emissions of the gas CCGT-CCS plant will be a function of the type and number of SUSD cycles. Consequently, the amount of CO_2_ removal required to reach net zero would vary, depending on how the plant is operated (e.g., hot SUSD vs. cold SUSD, number of SUSD cycles). Conversely, the residual emissions of blue hydrogen-CCGT do not increase with flexible operation. The ability of blue hydrogen-CCGT to operate flexibly without increasing its CO_2_ emissions intensity is highly advantageous. As the CO_2_ intensity remains relatively constant, the requirements of CO_2_ removal are predictable, which is favorable from an investment perspective as there is greater certainty on the cost of CO_2_ removal offsets.

This study highlights the importance of understanding the implications of constraints imposed by any sustainable green taxonomy requirements, which will likely dictate decisions and constrain parameters at both the plant and systems levels. Performing technology evaluations in the context of the sustainable taxonomy criteria are helpful in identifying approaches which are carbon inefficient, i.e., pathways that must be avoided, as well as promising pathways that align with the taxonomy criteria.

### Limitations of the study

There are some limitations to this study. The study analyses the CO_2_ intensity at a process-plant level for each different type of start-up and shut down, but does not demonstrate the combination of cold SUSD with hot SUSD. To understand the number and types of SUSD cycles that would occur in different regional electricity grids under specific scenarios, this would require energy systems optimization modeling, e.g., studies similar to de Mars, et al.^77^ Importantly, gas-fired CCGT power plants can vary in size between 500 and 2500 MW and operating mode (baseload vs. load following), with number of starts potentially ranging between 50–300.^77^^,^^78^ In this study, we assumed a 500 MW CCGT power plant with a generation efficiency of 58%_HHV_ (corresponds to unabated power plant) and the integration of CCS imposes an average energy penalty of 6.5% points.^79^^,^^80^^,^^81^ Although the absolute values may differ for CCGT plants of different generation capacity and efficiency, the relative trends and changes should be the same. For future work, energy systems modeling could be a useful tool to assess the role and value of hydrogen-fired CCGTs compared to NG-fired CCGTs as well as account for differences in plant capacity and efficiency.

Another important aspect to consider is the limitations of the downstream processes such as CO_2_ compression, which would require relatively stable CO_2_ flows despite the flexible operation and SUSD of the gas-fired CCGT-CCS plant. Thus, another advantage of the H_2_-CCGT system is that even with flexible operation of the power plant, the CO_2_ flows would likely be stable as hydrogen production operates at steady state.

## STAR★Methods

### Key resources table

**Table:** 

REAGENT or RESOURCE	SOURCE	IDENTIFIER
**Deposited data**		

Start-up and shut down plant data from the Technology Centre Mongstad (TCM) CO_2_ capture demonstration plant	IEAGHG Technical Report 2022-08 Start-up and Shutdown Protocol for Power Stations with CO_2_ Capture	https://ieaghg.org/ccs-resources/blog/new-ieaghg-report-2022-08-start-up-and-shutdown-protocol-for-power-stations-with-co2-capture
*Greenhouse gas reporting conversion factors*	Department for Business, Energy & Industrial Strategy (BEIS)	https://www.gov.uk/government/publications/greenhouse-gas-reporting-conversion-factors-2021
Supply emissions of British natural gas	Ecoinvent database V3	https://doi.org/10.1007/s11367-016-1087-8
Global average of supply emissions of natural gas	Sustainable Gas Institute, Imperial College London	https://www.imperial.ac.uk/media/imperial-college/research-centres-and-groups/sustainable-gas-institute/SGI_White_Paper_methane-and-CO2-emissions_WEB-FINAL.pdf
Life Cycle GHG Emission Study on the Use of LNG	Thinkstep	https://sea-lng.org/wp-content/uploads/2020/06/19-04-10_ts-SEA-LNG-and-SGMF-GHG-Analysis-of-LNG_Full_Report_v1.0.pdf
Power efficiency of natural gas-fired CCGT power plant with and without CCS	*Energy Procedia*	https://doi.org/10.1016/j.egypro.2011.02.122
Power efficiency of natural gas-fired CCGT power plant with and without CCS	Department for Business, Energy & Industrial Strategy (BEIS)	https://assets.publishing.service.gov.uk/government/uploads/system/uploads/attachment_data/file/911817/electricity-generation-cost-report-2020.pdf
CCS energy penalty (% points)	Energy Policy Research Group, University of Cambridge	https://www.jstor.org/stable/resrep30458?seq=1
CCS energy penalty (% points)	Department for Business, Energy & Industrial Strategy (BEIS)	https://assets.publishing.service.gov.uk/government/uploads/system/uploads/attachment_data/file/759538/2018_ESD_329.pdf

### Resource availability

#### Lead contact

Further information and requests for resources should be directed to and will be fulfilled by the lead contact, Niall Mac Dowell (niall@imperial.ac.uk).

#### Materials availability

This study did not generate new unique reagents.

### Method details

#### Method to evaluate CO_2_ emissions intensity

Using the natural gas supply chain emissions, assumptions and combustion data in the supplemental information, (Equation 1), (Equation 2), (Equation 3) are used to define the overall carbon footprint of blue hydrogen as a function of both the CO_2_ capture rate and the natural gas supply chain footprint:

(Equation 1) $$\begin{array}{c} dQ=A_{3}\times x \end{array}$$

(Equation 2) $$\begin{array}{c} A_{1}=A_{3}-dQ \end{array}$$

(Equation 3) $$\begin{array}{c} A_{2}=\frac{ER\times dQ}{\eta } \end{array}$$

where dQ is the amount of original emissions (i.e., grey hydrogen without CCS) avoided through CO_2_ capture, and x is the overall system CO_2_ capture rate, accounting for both the original emissions, and the emissions generated from the fuel supply used for the capture facility.

Both $A_{1}$ and $A_{2}$ (i.e., the residual emissions, and the natural gas requirement) are described as variables and are a function of the overall CO_2_ capture rate of the process.

$\frac{ER}{\eta }$ is the total natural gas requirement to avoid a given quantity of CO_2_, assumed to be a constant and composite parameter accounting for a boiler efficiency, η, of 90%.

Finally, $A_{1}$ and $A_{2}$ are combined with $A_{4}$ to provide an estimate of ${CI}_{blue\;hydrogen}$.

(Equation 4) $${CI}_{blue\;hydrogen}=A_{1}+\left(A_{2}+A_{4}\right)\times {CI}_{natural\;gas}$$

The model variables and parameters are as follows.

**Table:** 

**Variables** dQ [kg CO_2_,eq/MWh H_2_ LHV] x [-] $A_{1}$ [kg CO_2_,eq/MWh H_2_ LHV] $A_{2}$ [MWh natural gas LHV/MWh H_2_ LHV]
**Parameters** $\frac{ER}{\eta }$ (Natural gas requirement to avoid a given quantity of CO_2_) = 4 GJ/t CO_2_. $A_{3}$ (Residual emissions of grey hydrogen) = 269 kg CO_2_,eq/MWh H_2_ LHV.^82^ $A_{4}$ (Natural gas demand before CCS application) = 1.32 MWh natural gas LHV/ MWh H_2_ LHV

The variable bounds for CO_2_ capture rate, x, and carbon intensity of natural gas (i.e., supply chain emissions), ${CI}_{natural\;gas}$, are 80–100%, and 0–25 kg CO_2_,eq/GJ LHV, respectively.

The CO_2_ emissions intensity was calculated for a 500 MW combined cycle gas turbine (CCGT) power plant with a generation efficiency of 58%_HHV_ (corresponds to unabated power plant). The integration of CCS imposes an average energy penalty of 6.5% points.^79^^,^^80^^,^^81^ We also considered power plant electricity generation at steady state operation as well as flexible operation.

Two fuel scenarios were considered.

(1) Natural gas-fired CCGT with CCS (steady state and flexible operation);
(2) Blue hydrogen-fired CCGT, where hydrogen is produced from steam methane reforming (SMR) retrofitted with CCS (steady state operation only).

Importantly, the CO_2_ emissions intensity results in this study account for both Scope 1 (direct) and Scope 3 (indirect, i.e., associated with the supply chain) emissions.

#### Start-up and shut down calculations

For the flexible operation analysis, both cold and hot start-up and shut down cycles were considered, with the data used to describe these operating regimes shown in Table S4, supplemental information. Cold start-ups are typically performed after a long downtime (i.e., 8 hours or more), thus the stripper cools to near ambient temperature (25°C–40°C). In contrast, hot start-ups are performed after a short downtime (i.e., off for less than 8 hours), hence the stripper temperature is still high (e.g., 80°C or above). Thus, hot start-ups are typically quicker than cold start-ups.^44^

For hot start-up and shut down, the stripper bottom temperature remains hot at 80°C or above, and thus, does not need much energy to reach reboiler set-point temperature. The shut down procedure for the CO_2_ capture process includes the “lean out” of the entire solvent inventory prior to shut down, resulting in CO_2_ capture rates of higher than 99% for the next start-up as illustrated in Figure S4. The combination of these two effects results in high cumulative CO_2_ capture rates for the hot start-up and shut down cycles (Table S4). In contrast, the low ambient temperatures of cold start-ups require much more additional heating and time to bring the reboiler to the set-point temperature. Consequently, the cold start-up and shut down cycles have lower cumulative CO_2_ capture rates.

Over one year, the CCGT operation periods will be divided between periods of start-up and shut down, downtime and steady state operation. The period of steady state operation and the period of downtime over a year will be a function of the number, type and duration of start-up and shut down cycles. The power plant is assumed to generate electricity only during steady state periods. The CO_2_ emissions intensity of the natural gas-fired CCGT-CCS at different CO_2_ capture rates during steady state operation (i.e., with zero start-up and shut down cycles) is shown in Table S5. Three cases of steady state CO_2_ capture rates were assumed, 90%, 90% and 99%. The CO_2_ capture rate for different types of start-up and shut down cycles (cold vs hot, min vs max duration, and zero carbon intensity auxiliary boiler vs natural gas-fired auxiliary boiler) is summarised in Table S4, which simulates different configurations and operating modes (e.g., with or without CO_2_ capture of the emissions from the auxiliary boiler).

## Data Availability

The data that support the findings of this study are available from the lead contact upon reasonable request.
