# Supplementary material for: The prospects of flexible natural gas-fired CCGT within a green taxonomy
Source: iScience. 2023 Jul 14;26(8):107382. doi: 10.1016/j.isci.2023.107382 (PMC10407117; doi:10.1016/j.isci.2023.107382)
Supplement: Document S1. Figures S1–S5 and Tables S1–S6 [file mmc1.pdf]

iScience, Volume *26*

Supplemental information

The prospects of flexible natural gas-fired CCGT within a green taxonomy

Mai Bui, Nixon Sunny, and Niall Mac Dowell

Supplemental information

Natural gas supply chain emissions and combustion data

The assumption for Scope 1 Natural gas and Scope 3 supply chain emissions for different sources of gas are shown as vertical lines in Figure 3 and Figure 4 of the main paper.

Table S 1: Emissions intensity data of natural gas and references.

Parameter	Value	Reference
Scope 1 direct emissions of natural gas, i.e., via combustion (kg CO_2/GJ LHV)	56.64	BEIS [S1]
Scope 1 direct emissions of natural gas, i.e., via combustion (kg CO_2/tonnes NG)	2538.48	BEIS [S1]
Scope 3 emissions of British natural gas (kg CO_2/GJ LHV)	4.9	EcoInvent, Wernet, et al. [S2]
Global average of scope 3 emissions for natural gas (kg CO_2/GJ LHV)	14.9	Balcombe, et al. [S3]
Scope 3 emissions of LNG (kg CO_2/GJ LHV)	22.0	Schuller, et al. [S4]
Natural gas gross CV (MWh thermal/tonne NG)	13.859	BEIS [S1]

The scope 3 emissions of LNG (22.0 kg CO_2/GJ LHV) were taken from Schuller, et al. [S4], who used 30 for the GWP100 of methane (CH_4). These emissions are likely underestimated compared to a previous recommended value of 32.[S5]

Power plant assumptions

The CO_2 intensity of the CCGT power plant is calculated using the assumptions in Table S 2.

Table S 2: Assumptions used for the analysis of carbon intensity of the CCGT-CCS plant considered in this study.

Parameter	Assumption	References
Power generation capacity (MW)	500	
Capacity factor of a full-time plant	0.8	
Power plant efficiency unabated plant (% HHV)	58	Average of data from Popa, et al. [S6] and BEIS [S7]
Steady state capture rate (%)	90 or 99	
CCS energy penalty (% points)	6.5	Average of data from Popa [S8], Schnellmann, et al. [S9] and Bates and Read [S10]
Power plant efficiency with CCS energy penalty (%)	51.5	
UK supply chain carbon footprint of natural gas (kg CO_2eq/GJ LHV)	4.9	Wernet, et al. [S2]

TCM demonstration facility: Start-up and shut down plant data

A recent IEAGHG study[S11] quantified the CO_2 emissions associated with start-up and shut down of power plants with CO_2 capture. A series of start-up and shut down tests at the Technology Centre Mongstad (TCM) CO_2 capture facility, an industrial-scale post-combustion absorption plant, located near the Equinor oil refinery in Mongstad, Norway. The amine-based CO_2 absorption demonstration plant captures CO_2 from a natural gas-fired CCGT combined heat and power (CHP) plant using CESAR-1, an aqueous blend of AMP and PZ amines. Detailed analysis of the plant data was carried out to develop an understanding around the start-up and shut down (SUSD) dynamic behaviour. These results were used to inform the boundaries in term of CO_2 capture performance during start-up and shut down, summarised in Table S 4. For further information about this study, readers can refer to the detailed report published by IEAGHG.[S11]

Figure S 1 shows the start-up and shut down of the CO_2 capture plant in terms of some key process parameters: steam flow rate, inlet flue gas flow rate and lean solvent flow rate.

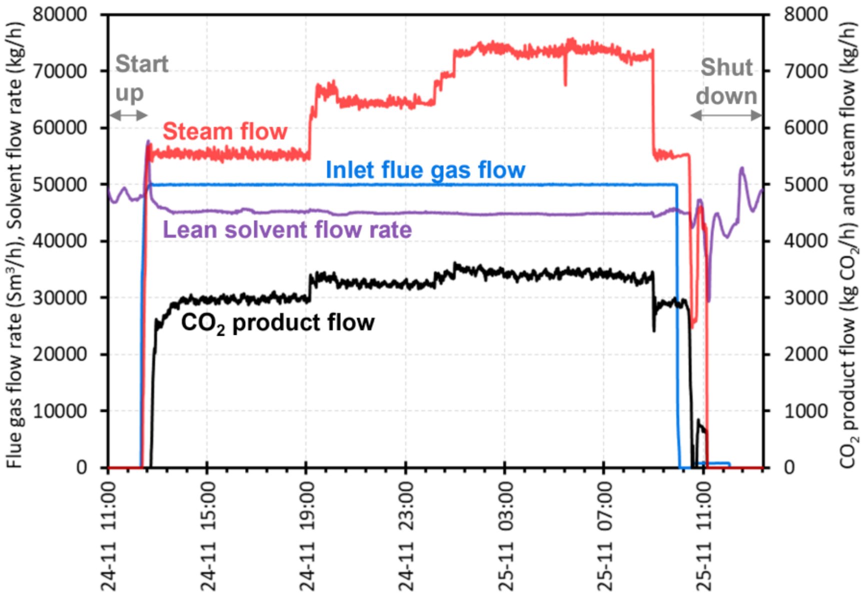

Figure S 1: Process parameter changes for a hot start-up and shut down of the Technology Centre Mongstad CO_2 capture facility in Norway using a solvent inventory of 53 m^3. Figure adapted from IEAGHG [S11].

A series of start-up and shut down tests were conducted to identify key process parameter that influence plant performance during:

1) **Cold start-up:** performed after a long downtime, i.e., >8 hours, stripper cools to near ambient temperature (25–40 °C). The time when CO_2 product flow starts is t_{SU}. Test above has steam flow starting at a similar time to the flue gas flow. Example shown in Figure S 2.

2) **Hot start-up:** performed after a short downtime, i.e., off for <8 hours, stripper is still high temperature at 80 °C or above. Thus, hot start-ups are much quicker than cold start-ups (shorter t_{SU}). Example shown in Figure S 3.

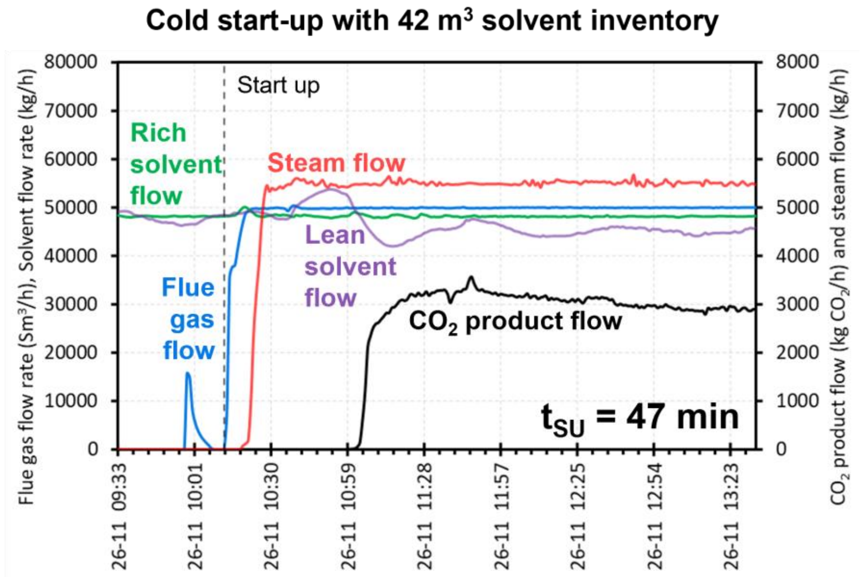

Figure S 2: Process parameter changes for cold start-up with a solvent inventory of 42 m^3 at the Technology Centre Mongstad CO_2 capture facility in Norway. Figure adapted from IEAGHG [S11]*. The start-up time t_{SU} of 47 min corresponds to the time it takes for the CO_2 product flow to start after flue gas flow begins.*

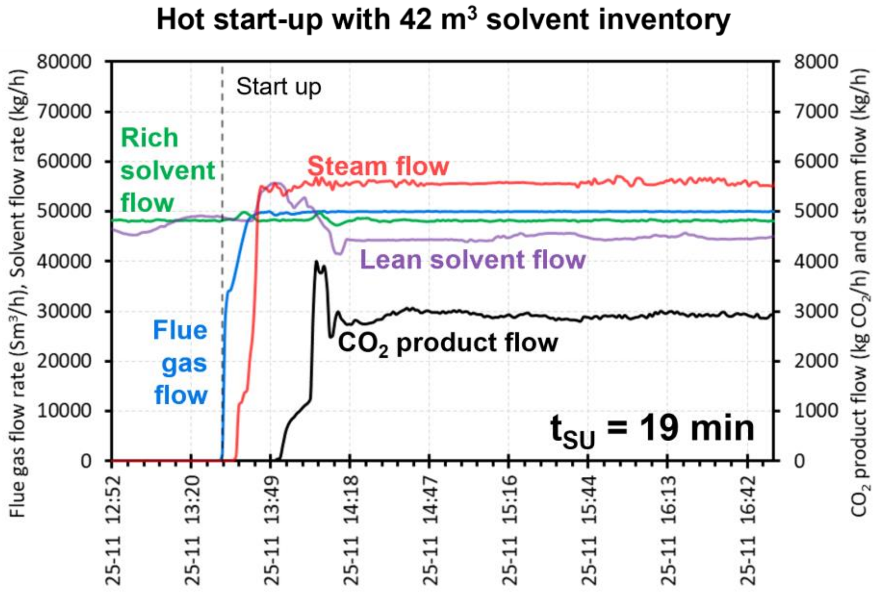

Figure S 3: Process parameter changes for hot start-up with a solvent inventory of 42m^3 at the Technology Centre Mongstad CO_2 capture facility in Norway. Figure adapted from IEAGHG [S11]*. The start-up time t_{SU} of 19 min corresponds to the time it takes for the CO_2 product flow to start after flue gas flow begins.*

The cold start-up simulates a start-up after a long downtime of power plant, where no steam is supplied to the CO_2 capture plant, and consequently, the reboiler bottom temperature has cooled to “ambient” conditions of 25–30°C. The hot start-up simulates a start-up after a short

downtime. As the power plant is only off for a short period (<8 hours), the CO_2 capture remains hot and the reboiler bottom temperature is ~90 °C.

The shut down period in Figure S 1 shows that steam supply to the CO_2 capture plant continues to supply after the flue gas was turned off to lean out the solvent until a target CO_2 loading is reached. By the end of shut down, the entire volume of solvent has low CO_2 loading (e.g., ≤ 0.2 mol CO_2/mol amine). Having lean solvent CO_2 loading for the next start-up will maximise the CO_2 capture capacity and achieve high CO_2 capture rates of 99% (as shown in Figure S 4). The approach of leaning out the solvent during shut down and starting up with low CO_2 loading aims to minimise residual CO_2 emissions associated with SUSD cycles. Therefore, this approach is also used in commercial-scale systems.[S12, 13]

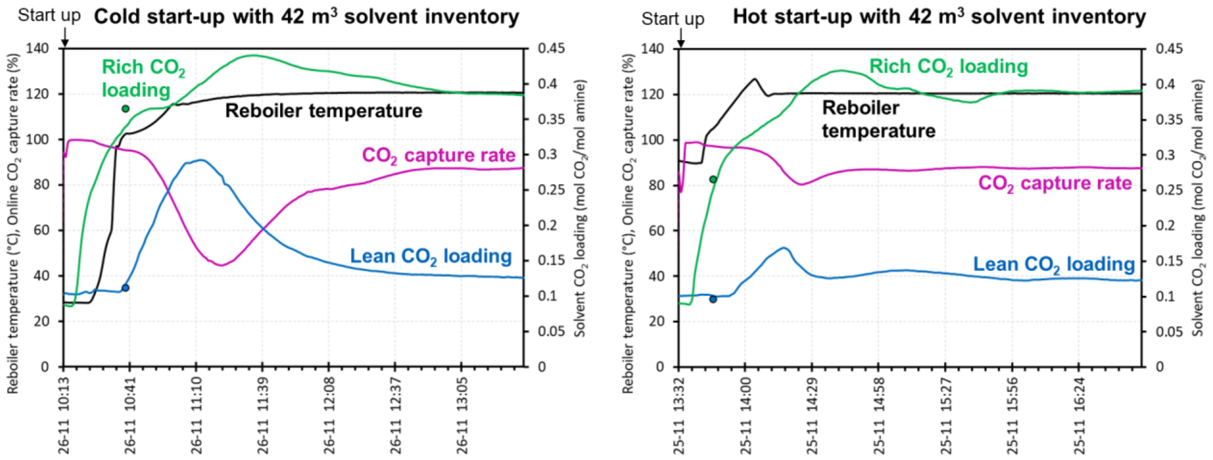

Figure S 4: Online CO_2 capture rate (purple), lean CO_2 loading (blue) and rich CO_2 loading (green) during cold start-up (Figure S 2) and hot start-up (Figure S 3).

Table S 3 shows the duration of a cold start-up is much longer than a hot start-up. As the CO_2 capture plant heats up during a cold start-up, the online CO_2 capture rate decreases from 99.8% to 44.6%, before increasing again to 87.6% (Figure S 4). For the hot start-up, the CO_2 capture rate starts at 98.8%, reduces slightly to 80.8% before reaching a steady state of 88.1%. Therefore, using hot start-ups provide a significantly higher cumulative CO_2 capture rate compared to cold start-ups (Table S 4).

Table S 3: Comparison of the cold and hot start-up tests in terms of start-up time (t_{SU}) and the time it takes for the system to reach steady state (t_{SS}).

Start-up type	Conditions	Start-up time: time when CO_2 product flow starts, t_{SU} (min)	Time when CO_2 product flow reaches steady state, t_{SS} (min)
Cold start-up	42 m^3 solvent inventory<br>Start loading 0.08–0.09 mol CO_2/mol amine	47	69
Hot start-up	42 m^3 solvent inventory<br>Start loading 0.08–0.09 mol CO_2/mol amine	19	42

Start-up and shut down calculations

Table S 4 presents the data for the duration of start-up and shut down cycles and the CO_2 capture rates, which is sourced from a recent study we published through IEAGHG.[S11] This IEAGHG study analysed the combined performance of start-up (SU) & shut down (SD) through real plant data from the TCM CO_2 capture facility. We assumed a natural gas-fired auxiliary boiler to generate heat/steam with an emissions factor of 50 kg CO_2/GJ.[S14]

For further detailed information and data on start-up and shut down tests conducted at the Technology Centre Mongstad (TCM) CO_2 capture plant, readers should refer the technical IEAGHG report.[S11]

Table S 4: Start-up and shut down data for time and CO_2 capture rate from IEAGHG [S11]. This data was used to calculate the CO_2 emissions intensity (Figure 6) of a natural gas CCGT-CCS plant as a function of the number of start-up and shut down cycles. There are two scenarios considered for the start-up and shut down duration: minimum time and maximum time, which result in different durations of time for requiring an auxiliary boiler.

**Start-up & shut down type**	**Duration of one start-up & shut down cycle (h)**	**Downtime (h)**	**Cumulative CO_2 capture rate with zero CO_2 intensity auxiliary boiler (%)**	**Cumulative CO_2 capture rate, accounts for NG CO_2 intensity of auxiliary boiler (%)**	**Duration of time using auxiliary boiler for SUSD (h)**
Cold start-up + shut down (Min time)	3	8	73.15	47.9	2
Cold start-up + shut down (Max time)	8	8	73.15	47.9	5
Hot start-up + shut down (Min time)	2	1	96.9	82.05	1.33
Hot start-up + shut down (Max time)	6	1	96.9	82.05	4.33

Table S 5: The CO_2 emissions intensity of the natural gas-fired CCGT-CCS at different CO_2 capture rates during steady state operation (i.e., with zero SUSD cycles).

	**CO_2 capture rate**		
	**90%**	**95%**	**99%**
**CO_2 emissions intensity (kg CO_2 eq/MWh_{el})**	75.2	53.2	35.5

In a given year, the period of steady state operation will be a function of the number, type and duration of start-up and shut down cycles. The power plant is assumed to generate electricity only during steady state periods. As the number of start-up and shut down cycles increases, the period of steady state operation reduces, and the capacity factor of the power plant reduces, reducing the power generation. During periods of steady state operation, the CO_2 capture rate is assumed to be constant, either 90%, 95% (Figure 6) or 99% (Figure S 5).

Table S 6: Number of start-up and shut down cycles permitted per year for a natural gas-fired CCGT-CCS within a sustainable taxonomy that has a CO_2 emissions intensity threshold of 100 kg CO_2 eq/MWh_{el} (data from Figure 6 and Figure S 5).

	At CO_2 emissions intensity of 100 kg CO_2 eq/MWh_{el}: **Number of SUSD cycles per year**					
	**90% capture during steady state**		**95% capture during steady state**		**99% capture during steady state**	
	**NG auxiliary boiler**	**Zero carbon auxiliary boiler**	**NG auxiliary boiler**	**Zero carbon auxiliary boiler**	**NG auxiliary boiler**	**Zero carbon auxiliary boiler**
**Cold SUSD (min duration)**	221	311	328	No limit	No limit	No limit
**Cold SUSD (max duration)**	102	153	166	232	205	275
**Hot SUSD (min duration)**	No limit	No limit	No limit	No limit	No limit	No limit
**Hot SUSD (max duration)**	291	No limit	No limit	No limit	No limit	No limit

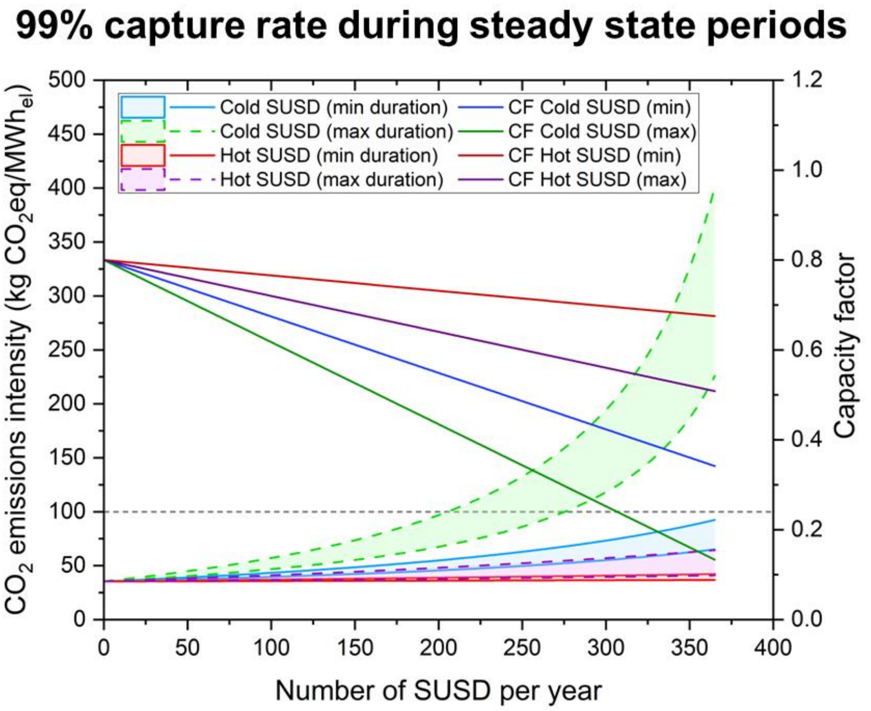

Figure S 5: Effect of increasing start-up and shut down (SUSD) cycles on the CO_2 emissions intensity and capacity factor of a CCGT power plant with CCS, assuming 99% capture rate. The CO_2 emissions intensity of the plant is a function of type of start-up (cold vs hot) and the duration(min vs max times). The CO_2 emissions intensity account for both Scope 1 (direct combustion) and Scope 3 (supply chain) emissions.

References

S1. BEIS, (2020), *Greenhouse gas reporting: conversion factors 2021*, Department for Business, Energy & Industrial Strategy, UK, https://www.gov.uk/government/publications/greenhouse-gas-reporting-conversion-factors-2021.

S2. G. Wernet, C. Bauer, B. Steubing, J. Reinhard, E. Moreno-Ruiz and B. Weidema, (2016), The ecoinvent database version 3 (part I): overview and methodology. *The International Journal of Life Cycle Assessment*, **21**, 1218-1230. 10.1007/s11367-016-1087-8

S3. P. Balcombe, K. Anderson, J. Speirs, N. Brandon and H. Adam, (2015), *Methane & CO_2 emissions from the natural gas supply chain report*, Sustainable Gas Institute, Imperial College London, https://www.imperial.ac.uk/media/imperial-college/research-centres-and-groups/sustainable-gas-institute/SGI_White_Paper_methane-and-CO2-emissions_WEB-FINAL.pdf.

S4. O. Schuller, S. Kupferschmid, J. Hengstler and S. Whitehouse, (2019), *Life Cycle GHG Emission Study on the Use of LNG as Marine Fuel Final Report*, Thinkstep, https://sea-lng.org/wp-content/uploads/2020/06/19-04-10_ts-SEA-LNG-and-SGMF-GHG-Analysis-of-LNG_Full_Report_v1.0.pdf.

S5. C. D. Holmes, M. J. Prather, O. A. Søvde and G. Myhre, (2013), Future methane, hydroxyl, and their uncertainties: key climate and emission parameters for future predictions. *Atmos. Chem. Phys.*, **13**, 285-302. 10.5194/acp-13-285-2013

S6. A. Popa, R. Edwards and I. Aandi, (2011), Carbon capture considerations for combined cycle gas turbine. *Energy Procedia*, **4**, 2315-2323. https://doi.org/10.1016/j.egypro.2011.02.122

S7. BEIS, (2020), *Electricity generation costs 2020*, Department for Business, Energy & Industrial Strategy, https://assets.publishing.service.gov.uk/government/uploads/system/uploads/attachment_data/file/911817/electricity-generation-cost-report-2020.pdf.

S8. A. Popa, (2011), *CCGT with CCS - integration options*, 1st Post Combustion Capture Conference, 17-19 May 2011, Abu Dhabi, IEAGHG, https://ieaghg.org/docs/General_Docs/PCCC1/Abstracts_Final/pccc1Abstract00055.pdf.

S9. M. A. Schnellmann, C. K. Chyong, D. M. Reiner and S. A. Scott, (2018), *Deploying gas power with CCS: The role of operational flexibility, merit order and the future energy system*, Energy Policy Research Group, University of Cambridge, https://www.jstor.org/stable/resrep30458?seq=1.
S10. C. Bates and A. Read, (2018), *BEIS: CCUS Technical Advisory – Report on Assumptions*, Uniper Technologies, https://assets.publishing.service.gov.uk/government/uploads/system/uploads/attachment_data/file/759538/2018_ESD_329.pdf.
S11. IEAGHG, (2022), *Start-up and shutdown protocol for natural gas-fired power stations with CO_2 capture, Report 2022-08, authors: Bui, M., Mac Dowell, N., Campbell, M., Knarvik, A. B. N.*, IEAGHG, United Kingdom.
S12. Bechtel, (2009), *CO_2 capture facility at Kårstø, Norway - Front-End Engineering and Design (FEED) Study Report. 320-CCC FEED Study*, Bechtel Power Corporation, https://ukccsrc.ac.uk/wp-content/uploads/2020/03/8.5-x-11-Full-Karsto-FEED-Study-Report-Redacted-Updated_OCR-1.pdf.
S13. Bechtel, (2009), *Operating and Maintenance Philosophy - CO_2 capture facility, Kårstø, Norway*, Bechtel Power Corporation, https://ukccsrc.ac.uk/wp-content/uploads/2020/03/Operating-and-Maintenance-Philosophy-25474-000-517-U07G-00001r001v2_0.pdf.
S14. BEIS, (2019), *2019 Government greenhouse gas conversion factors for company reporting: Methodology paper for emission factors final report*, Department for Business, Energy & Industrial Strategy, https://assets.publishing.service.gov.uk/government/uploads/system/uploads/attachment_data/file/904215/2019-ghg-conversion-factors-methodology-v01-02.pdf.
